# How Does Alkali Aid Protein Extraction in Green Tea Leaf Residue: A Basis for Integrated Biorefinery of Leaves

**DOI:** 10.1371/journal.pone.0133046

**Published:** 2015-07-22

**Authors:** Chen Zhang, Johan P. M. Sanders, Ting T. Xiao, Marieke E. Bruins

**Affiliations:** 1 Biobased Chemistry and Technology Group, AFSG, Bornse Weilanden 9, 6708WG Wageningen, Wageningen UR, the Netherlands; 2 Food and Biobased Research Institute, Bornse Weilanden 9, 6708WG Wageningen, Wageningen UR, the Netherlands; 3 Department of Plant Sciences, Laboratory of Molecular Biology, Droevendaalsesteeg 1, 6708 PB, Wageningen, Wageningen UR, the Netherlands; Old Dominion Univ., UNITED STATES

## Abstract

Leaf protein can be obtained cost-efficiently by alkaline extraction, but overuse of chemicals and low quality of (denatured) protein limits its application. The research objective was to investigate how alkali aids protein extraction of green tea leaf residue, and use these results for further improvements in alkaline protein biorefinery. Protein extraction yield was studied for correlation to morphology of leaf tissue structure, protein solubility and hydrolysis degree, and yields of non-protein components obtained at various conditions. Alkaline protein extraction was not facilitated by increased solubility or hydrolysis of protein, but positively correlated to leaf tissue disruption. HG pectin, RGII pectin, and organic acids were extracted before protein extraction, which was followed by the extraction of cellulose and hemi-cellulose. RGI pectin and lignin were both linear to protein yield. The yields of these two components were 80% and 25% respectively when 95% protein was extracted, which indicated that RGI pectin is more likely to be the key limitation to leaf protein extraction. An integrated biorefinery was designed based on these results.

## Introduction

Leaf protein has been considered as an additional protein source since 1960s [1, 2]. These proteins can be used in food [3], animal feed [4], or when hydrolysed to amino acids for N-chemicals bulk chemicals [5]. However, applications of these proteins are limited by its low cost-efficient production [6], particularly in extraction processes. This limitation was preliminarily solved by using alkaline conditions at higher than 60°C [7]. Drawbacks of this technique are overuse of chemicals and low quality of (denatured) protein. To overcome these drawbacks and design an integrated process for protein and other products, the basis of alkaline protein extraction in leaf should be better understood.

Alkali might aid leaf protein extraction as a result of leaf tissue disruption. Leaf has three major tissue systems: epidermis, mesophyll, and vascular. Vascular tissues are located in mesophyll tissues covered by epidermis tissue. Those tissues are adhered by lamella layer embracing a large quantity of pectin. Epidermis is a tabular and layered sheet of cells on surface of leaf covered by a waxy cuticle functioning as mechanical protection of mesophyll tissue [8, 9]. As most leaf proteins, including lectins, enzymes (Rubisco), storage proteins, cell wall proteins and some toxins [10] are located in mesophyll tissues [8, 11], disruption of epidermis and lamella might aid protein release from mesophyll tissues.

Alkali might also aid leaf protein extraction by increasing protein solubility or / and hydrolysis degree. Most leaf proteins are considered insoluble [12–14]. These protein are hydrophobic in neutral solution, because they are bound to other compounds, such as polyphenol [15] or polysaccharides (membrane protein) [16]. Solubility of these proteins can be increased with the increase of pH by adding alkali. At high temperature, alkali can even hydrolyse protein into small peptides [17, 18], which reduces protein molecular size, and therefore increases protein solubility and accelerates protein diffusion.

Finally, alkali might aid in disruption of cell wall and thus leading to high protein yield. Leaf proteins within cells are well protected by the cell walls, which consist of middle lamella, primary wall, and secondary wall [8]. In cell wall, carbohydrates (including pectin, hemi-cellulose, and cellulose) and lignin are two major components other than protein [8]. Pectin is a family of complex polysaccharides located in primary plant cell wall and middle lamella [8, 19]. It can be roughly divided into three types: homogalacturonan (HG), rhamnogalacturonan I (RGI), and rhamnogalacturonan II (RGII) [20]. Hemi-cellulose and cellulose are mainly found in both primary and secondary plant cell wall, and they have a simpler composition than pectin. In comparison, lignin could be the most complicated component located in secondary plant cell wall [8]. It is a complex phenolic polymer that drives out water and strengthens the cell wall [8]. Solubilisation of carbohydrates and lignin performs differently under different alkaline conditions [21–24], therefore correlating yields of these components with protein yield may profile how alkali disrupts cell wall, and offer a basis for integrated leaf biorefinery.

Green tea residue (GTR) is used as a model material for research on leaf biorefinery. It was demonstrated earlier that the concept of protein extraction using GTR as a model material can also be applied in Oolong tea leaf residue, Jatropha leaf, barley straw [7], and even on algae. GTR is the waste of tea leaves after hot water extraction, containing high value components, such as polyphenols (10–15%), proteins (20–30%), and carbohydrates (30–40%) [25, 26]. It is now only used for energy generation through burning. An integrated biorefinery targeting on high value components of GTR will increase its value.

In this study, GTR was again used as a model material, and protein extracts were obtained at various alkaline extraction conditions based on our previous work [7]. Alkaline treated leaf tissues were analysed by microscope; solubility and hydrolysis degree of extracted protein and the composition of extracted carbohydrates was determined; extracted amounts of lignin were estimated. These results were all correlated to protein yield to analyse how alkali aids protein extraction in GTR. Based on these results, potential of integrated biorefinery of protein and other components for leaves was discussed.

## Methods and Materials

### Ethics statement

“N/A”.

No specific permissions were required for these locations/activities because we used local species or species present naturally in our environment. We confirm that the field studies did not involve endangered or protected species. We had the permission for the research on this green tea residue from Damin Company.

### Materials

Green tea residue (GTR) is our main material, which is a gift from Damin Company, Fujian Province, China. This residue from tea lemonade production was collected from *C*. *sinensis* trees in Zhejiang province, and it was sun-dried after soaking green tea leaves in water at 85°C for 45min. Chemicals used for analysis were purchased from Sigma (USA, analytic grade) if not stated otherwise.

### Preparation of alkaline extracts

Protein extraction was performed by soaking 0.5g GTR in 20ml 0.1M NaOH at 25°C, 60°C, and 95°C for 2h. After subsequent centrifugation, which was always performed at 15000g for 10min (Sorvall centrifuge, Thermo Fisher Scientific, the USA), the supernatants were then stored at -20°C for further analysis. The corresponding solid residues were collected and washed with water for 3 times, and then they were immersed in water and analysed by microscopy immediately.

For analysing the correlations of carbohydrates or lignin with protein, samples were made by soaking 0.5g GTR in 20ml 0.1M NaOH at 25°C, 60°C, and 95°C over time (5min-24h). Samples were freeze-dried and stored at room temperature till further use.

### Visualization of leaf tissues

Solid samples obtained after alkaline treatment for 2h, as well as a control of untreated material and a control treated by 0.05M NaOH, were examined under the microscope (SMZ-U, Nikon, Japan). Pictures were taken with a BCE-C050 Camara (Mightex, US) that was fitted on to the microscope.

### Determination of protein properties

#### Protein content of extracts

Protein content (g L^-1^) was determined by Kjeldahl method [27], using Kjeldahl equipment from Gerhardt, which consist of digestion unit (Gerhardt Kjeldahlterm) and rapid distillation unit (Gerhardt Vapodest). Kjeldahl measures all nitrogen, including non-protein N-containing components, such as caffeine, chlorophyll, and theobromine [28]. However, we assumed that it is all protein and used a conversion factor of 6.25 to calculate protein concentrations. Protein extraction yield was calculated as extracted protein / total protein * 100%.

#### Protein hydrolysis degree

Protein hydrolysis degree is defined as the percentage of cleaved peptides bonds. As the amount of fully hydrolysed protein from GTR is the same for each sample, here we use the difference of-NH_2_ residue before and after hydrolysis to discuss the hydrolysis degree. The content of-NH_2_ residue was determined by a modified o-phthaldialdehyde (OPA) method [29]. 1.5ml OPA reagent (OPA 0.88g L^-1^, dithiothreitol 0.88g L^-1^, SDS 1g L^-1^, and Na_2_B_4_O_7_·10H_2_O 38.1g L^-1^) was mixed with 200μl serine (0.1g L^-1^), sample, or water (as a blank). After exactly 2 min, absorbance of the mixture was determined spectrophotometrically at 340nm. Concentration of-NH_2_ residue was calculated using serine as a reference.

#### Protein solubility

Alkaline extracts were diluted to a protein concentration of 10g L^-1^. Protein solutions pHs were adjusted to 2–11 using 0.5 M HCl, after which the solution was kept stirring at room temperature for 1 h. Samples were subsequently centrifuged at 15000g for 10min at room temperature and the supernatant was collected. The amount of soluble protein in the filtrate was determined by Lowry method (Sigma, Lowry total protein determination kit) using bovine serum albumin as standard. Solubility is presented as a percentage of total protein in weight.

### Water and ash content

Samples were pre-weight and dried for 24 hours at 60°C to evaporate all water. After cooling down in a desiccator, the residual weights of samples were measured. Then, samples were transferred to a 550°C furnace for 16h to burn off all the organic matter. After cooling in a desiccator, the crucibles were weighed. Water content and ash content were calculated as weight percentage of the starting material.

### Polyphenol content

Content of tea polyphenols in tea extracts was determined spectrophotometrically with scaling down reagent usage [30, 31]. Polyphenols content was calculated assuming a concentration of polyphenols of 3.914g L^-1^leads to an adsorption of 1 at 540 nm after reaction.

### Galacturonic acid determination

Galacturonic acid content was determined as anhydro-uronic acid by an automated m-hydroxydiphenyl assay with an auto-analyzer (Skalar Analytical BV, Breda, The Netherlands) [32]. Galacturonic acid (Fluka AG, Buchs, Switzerland) was used as a reference in a concentration range from 12.5 to 200 mg L^−1^.

### Neutral sugar composition

Freeze dried alkaline extracts were pre-hydrolysed with 72% (w/w) sulphuric acid at 39°C for 1 h, followed by hydrolysis in 1 M sulphuric acid at 100°C for 3 h. Monosaccharides were analysed using GC according to Englyst’s method [33]. Inositol was used as internal standard. Response factor was determined using a standard sugar solution of L-(+)-rhamnose, L-(+)-arabinose, D-(+)-xylose, D-(+)-mannose, D-(+)-galactose (97%), D-(+)-glucose (99,5%) with concentrations of 1 g L^-1^.

To quantify carbohydrates, indirect assays are often used. Samples are hydrolysed to mono-sugars, and these sugars are quantified by HPLC or GC [33].HG pectin consists only of galacturonic acid of which some of the carboxyl groups are methyl esterified [20, 34].HG pectin can be analysed by galacturonic acid content. RGI consists of a backbone of repeating disaccharide of galacturonic acid and rhamnose. A variety of different glycan chains (principally arabinan and galactan) are attached to the rhamnose residues. As RGI pectin is predominated by side chains [20, 34] mainly constituted from arabinose and galactose, it can be analysed by the contents of these two mono-sugars. In comparison, RGII has a backbone of HG with complex side chains attached to the galacturonic acid [20, 34]. Therefore RGII analysis needs information of all sugars contents. Hemi-cellulose’s backbone comprises of xylose and glucose, while cellulose is a linear chain of glucose [23, 24].

All the experiments were performed in duplicate, and all results were plotted in the figures.

## Results

### Influence of alkali on leaf tissues

**Fig 1:**
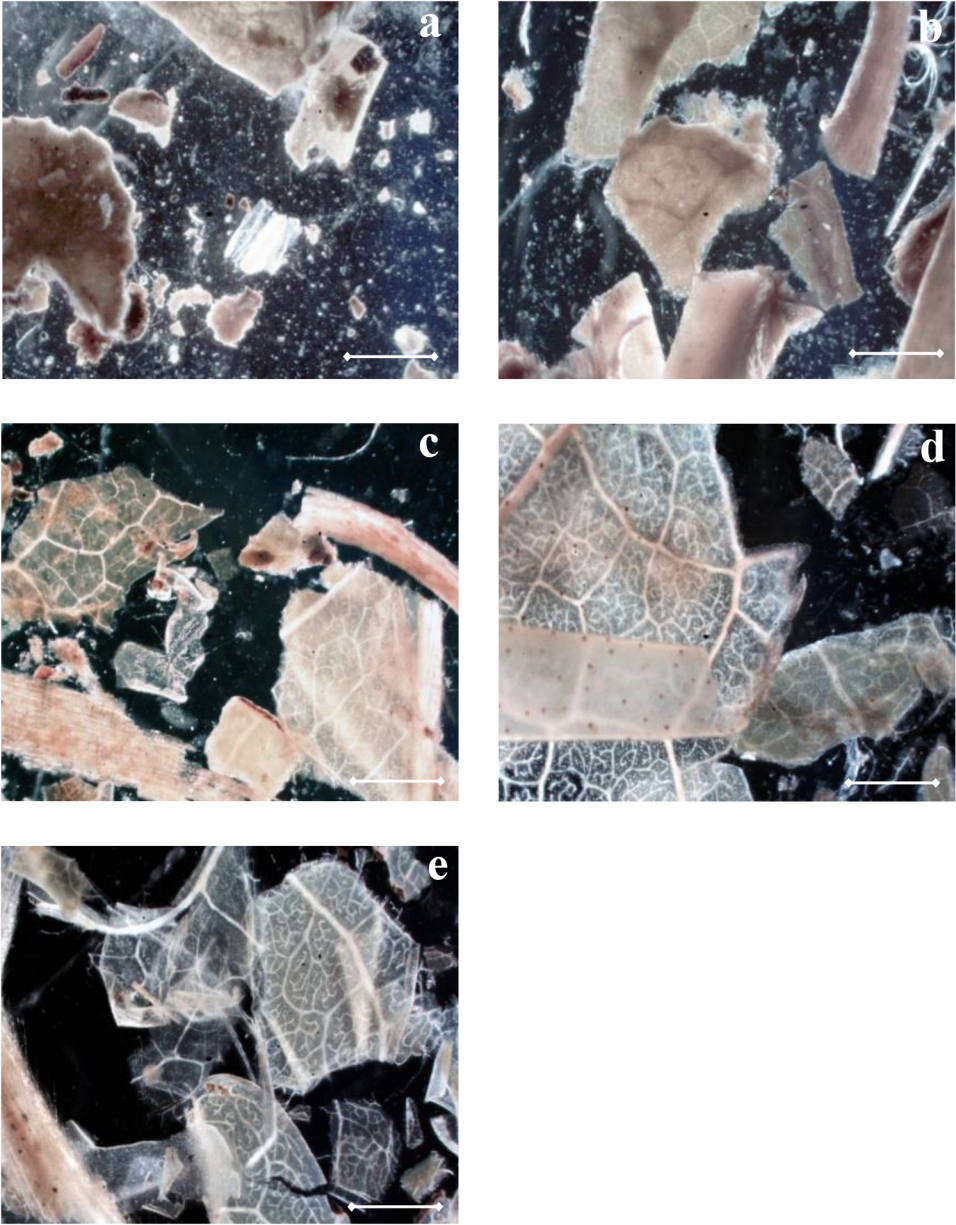
Morphology of GTR tissues after a 2h treatment with 40 v/w solution (scale bar: 100μm). a. GTR treated with water at 25°C. b. GTR treated with 0.05M NaOH at 95°C. c. GTR treated with 0.1M NaOH at 25°C. d. GTR treated with 0.1M NaOH at 60°C. e. GTR treated with 0.1M NaOH at 95°C.

As mentioned, leaf consists of three major tissues systems adhered by middle lamella. To study the disruption of leaf tissues under alkaline conditions, alkali treated GTRs were analysed microscopically. Although GTR is the leftover of green tea leaves treated by hot water, the structure of its tissue is still intact. Shown in Fig 1A, the epidermal layer of untreated GTR was normally attached to the mesophyll tissue and the lamella was not solubilized, shown as a low transparency of leaf tissue and visible fragments suspended in the solution. After treatment with 0.1M NaOH, leaf tissues were transparent and solutions became clear (Fig 1C, 1D and 1E). At 25°C, the epidermal layer was still attached to the mesophyll tissue (Fig 1C), but it started to peel off when higher temperature was applied (Fig 1D and 1E). As protein yield increased with the increase of temperature (23% obtained by 25°C, 38% obtained by 60°C, and 84% obtained by 95°C), these figures indicate a correlation of leaf tissue disruption with protein yield under alkaline conditions.

When alkaline concentration was limited to 0.05M, GTR tissues (Fig 1B) are similar as the untreated one (Fig 1A). The protein yield obtained by using 0.05M NaOH at 95°C was about 40% [7], which was similar to the yield obtained by 0.1M NaOH at 60°C (38%). However, the transparency of the tissue treated with 0.05M NaOH was lower than that treated by 0.1M NaOH. This indicates that alkali was used to solubilize substances between cells, while high temperature was related to protein located inside cells or membrane, which is commonly identified as insoluble [16]. To further understand how does alkali aids in protein extraction, influence of alkali on protein hydrolysis and solubility was tested, and yields of non-protein components were subsequently analysed and correlated to protein yields.

### Composition of GTR

To calculate yields of all components in alkaline condition, composition of GTR was analysed. Carbohydrate and protein (or more accurate: all N-containing components) are the two major components, accounting for 31% and 27% of GTR. Other components are polyphenol (8%), water (7%), and ash (6%). The residual undetermined part majorly consists of lipid (wax, organic acids) and lignin [35]. As lipid and lignin will not be extracted by the pre-treatment with hot water that was done in the factory, the ratio of lipid to lignin (2:6.5) presented in green tea leaf [35] is estimated to remain constant in GTR, leading to 5% and 17% of dry GTR respectively. Carbohydrates were quantified by summing all mono-sugars. Glucose and galacturonic acid are the major sugar components constituting 13.4% and 7.6% of GTR, followed by galactose, arabinose, and xylose with percentages of 3.4%, 2.8%, and 2.1% of GTR respectively. Other less abundant sugars are mannose with 1.1%, rhamnose with 0.8%, and fucose with 0.4%.

### Influence of alkali on protein hydrolysis and solubility

Generally, 1g native protein already contains 0.342–0.457 mmol-NH_2_ groups, when protein is fully hydrolysed,-NH_2_ content will increase to 8.6 mmol depending on amino acid composition [7, 29]. To compare hydrolysis degree, protein extracts obtained using 0.1M NaOH with v/w of 40 for 2h at 25°C, 60°C, and 95°C were tested by OPA method. The protein yields were 23% at 25°C, 38% at 60°C, and 84% at 95°C. In these samples,-NH_2_ content in 1g protein was 0.43 ± 0.03 mmol, 0.54 ± 0.07 mmol, 0.19 ± 0.02 mmol respectively. Those numbers are close to the value of native protein, suggesting that only very limited hydrolysis occurred. When 95°C was applied, the-NH_2_ content was even reduced. This may result from reactions of-NH_2_ with other components, such as polyphenol [36]. Protein hydrolysis degree did not correlate with protein yield.

**Fig 2:**
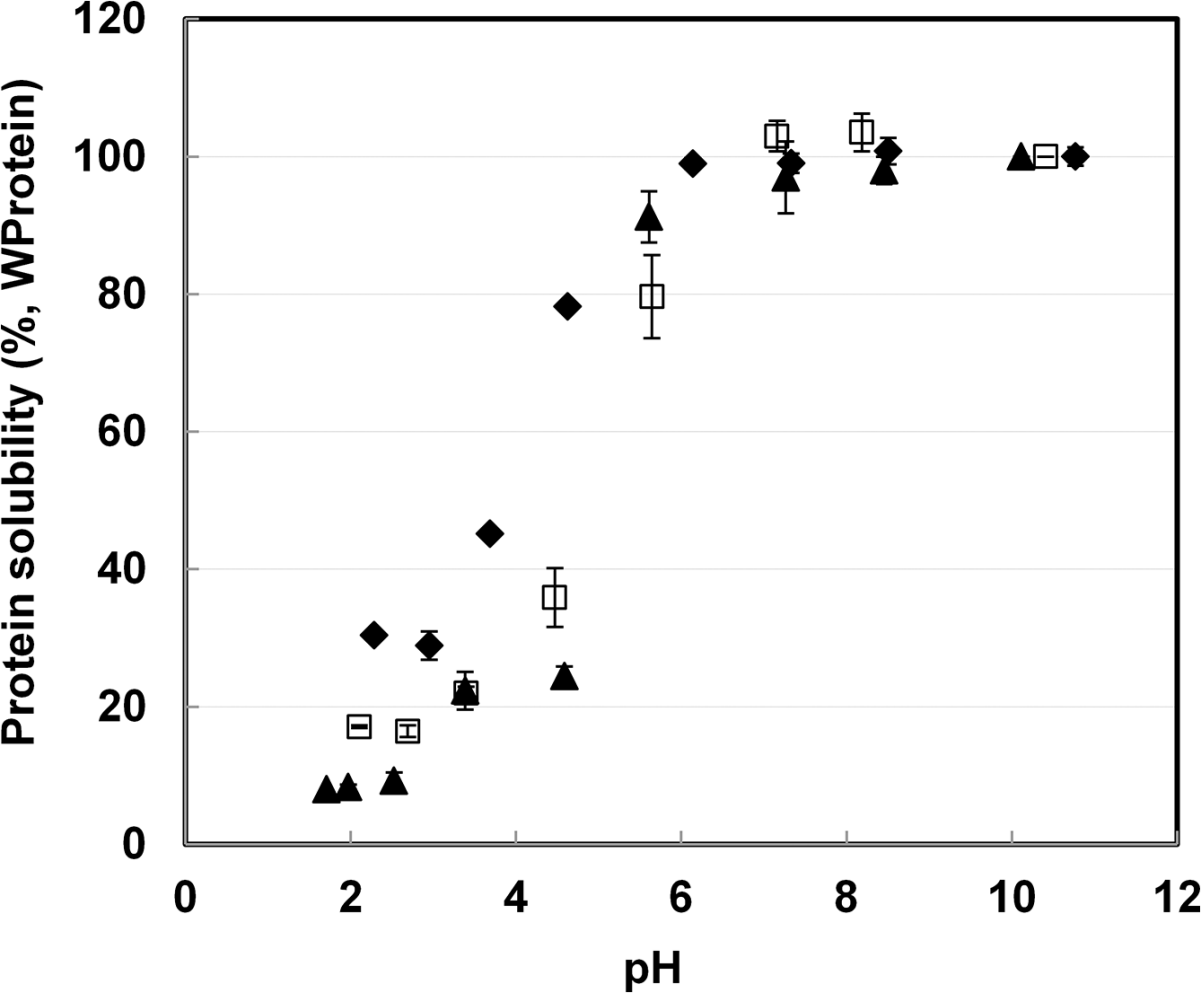
Solubility of protein extract as a function of pH obtained from experiments using 0.1M NaOH with v/w of 40 for 2h at different temperatures. ◆: 25°C;: **□** 60°C; ▲:95°C.

To determine the correlation of protein solubility with protein yield, protein solubility was measured as a function of pH. Samples from three temperatures, as mentioned above, were used and the result is depicted in Fig 2. At pH higher than 6, protein solubility of all samples remained at the maximal, added amount of 10g L^-1^. This is over twice the amount of extracts from other research [26, 37]. Based on these results, we conclude that protein solubility was not a limitation to protein yield. Below pH 6, solubility of all three protein extracts decreased. The 95°C sample had its lowest protein solubility at pH<4.5. In other cases solubility of hydrolysed protein increased between pH 2–6 when more severe hydrolysis had happened [17, 18]. This again indicates that protein was not severely hydrolysed after alkaline treatment at 95°C. Alkaline protein extraction is therefore not facilitated by hydrolysis or increased solubility of protein.

### Correlation of carbohydrates yields with protein yields

#### Pectin

**Fig 3:**
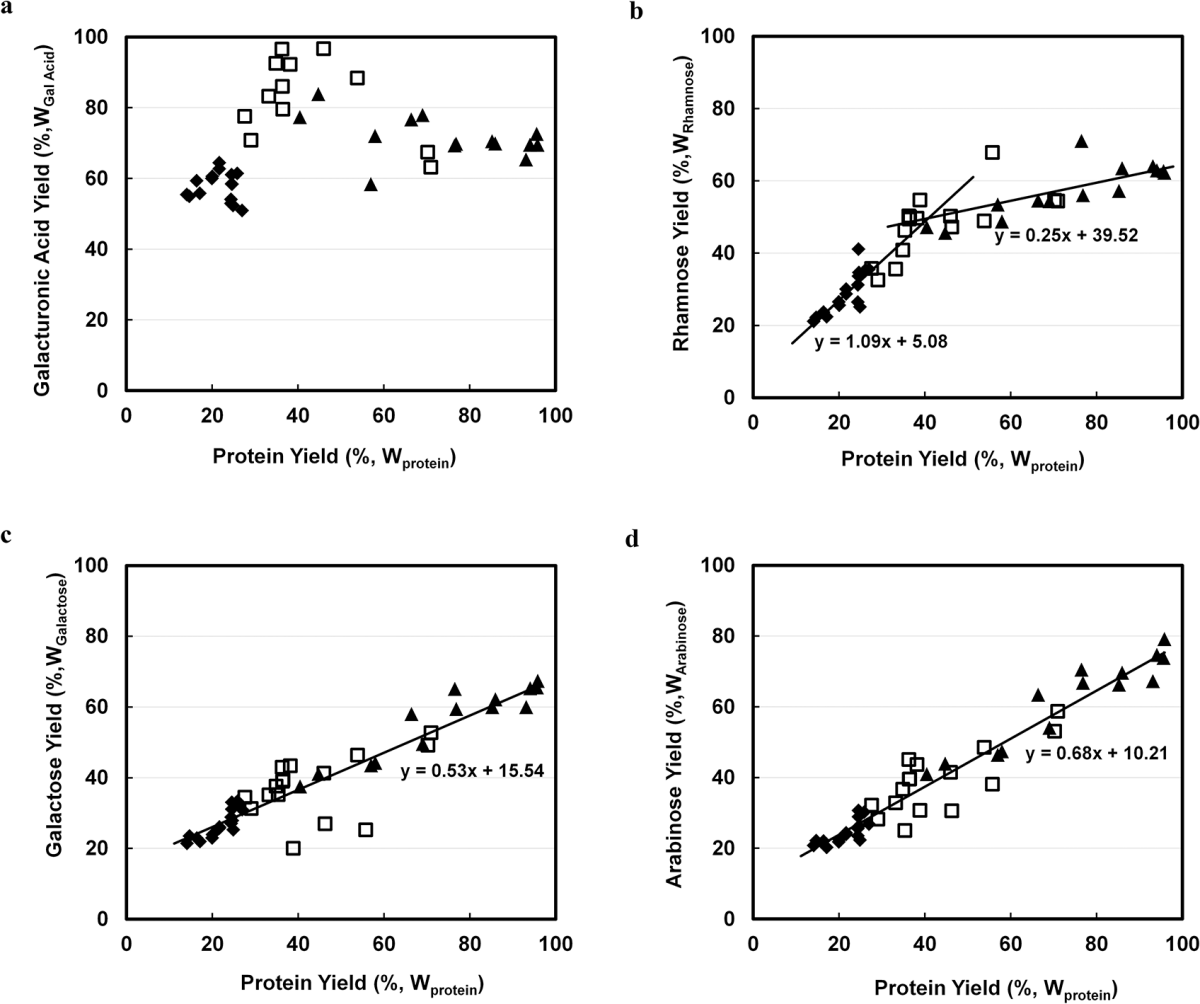
Weight based correlation of extracted pectin related sugars with extracted protein (%,W_Protein_) by using 0.1M NaOH with 40 v/w at ◆: 25; □: 60°C; ▲ 95°C. a. Galacturonic acid (%,W_**Galacturonic Acid**_). b. Rhamnose (%,W_**Rhamnose**_). c. Galactose (%,W_**Galactose**_). d. Arabinose (%,W_**Arabinose**_).

As mentioned in introduction, galacturonic acid, rhamnose, galactose, and arabinose can be used to analyse pectin yields and types. Yields of galacturonic acid, rhamnose, galactose, and arabinose in protein extracts were plotted against protein yields in Fig 3.

At mild conditions (all 25°C samples and samples of 60°C treated less than 4h), yields of galacturonic acid were positively correlated with protein yield. Galacturonic acid yield was highest at 95% when around 40% of protein was extracted (Fig 3A), which occurred at 60°C, after 2–4 hours of extraction. When higher temperature was applied (95°C), galacturonic acid yield was less. This lower yield might result from the β-elimination of galacturonic acid under harsh alkaline conditions [38]. As HG pectin consists of only galacturonic acids, HG pectin was suggested to be completely extracted at relatively mild conditions. Therefore, HG pectin is not the limitation for obtaining high protein yield.

Rhamnose yields were also positively correlated to protein yields, but a bend was observed in the curve in Fig 3B and two different lines and slopes were obtained. The coefficient of rhamnose yield to protein yield was 1.09 when less than 40% of protein was extracted, and their coefficient was 0.25 when more than 40% protein was extracted. The two coefficients indicate that rhamnose may originate from two pectin sources, RGI and RGII, and one of them might be already completely extracted at mild conditions.

Yields of galactose and arabinose were linear to protein yields throughout the entire time and temperature range (Fig 3C & 3D). When 95% protein was extracted, the yields of galactose and arabinose were about 70% and 80% respectively. The coefficient of galactose yield to protein yield was 0.53, while the coefficient of arabinose yield to protein yield was 0.68. As galactose and arabinose majorly originate from RGI pectin [20, 34], these results indicate a linearity of RGI pectin yield to protein yield, and RGI may therefore be the limitation to protein extraction. Combined with the result of the correlation on rhamnose yield to protein yield, RGII pectin was probably completely extracted at mild conditions. This conclusion could be confirmed by another study, in which RGII of green tea leaf was shown to be more soluble than RGI in water [39].

#### Hemi-cellulose& cellulose

**Fig 4:**
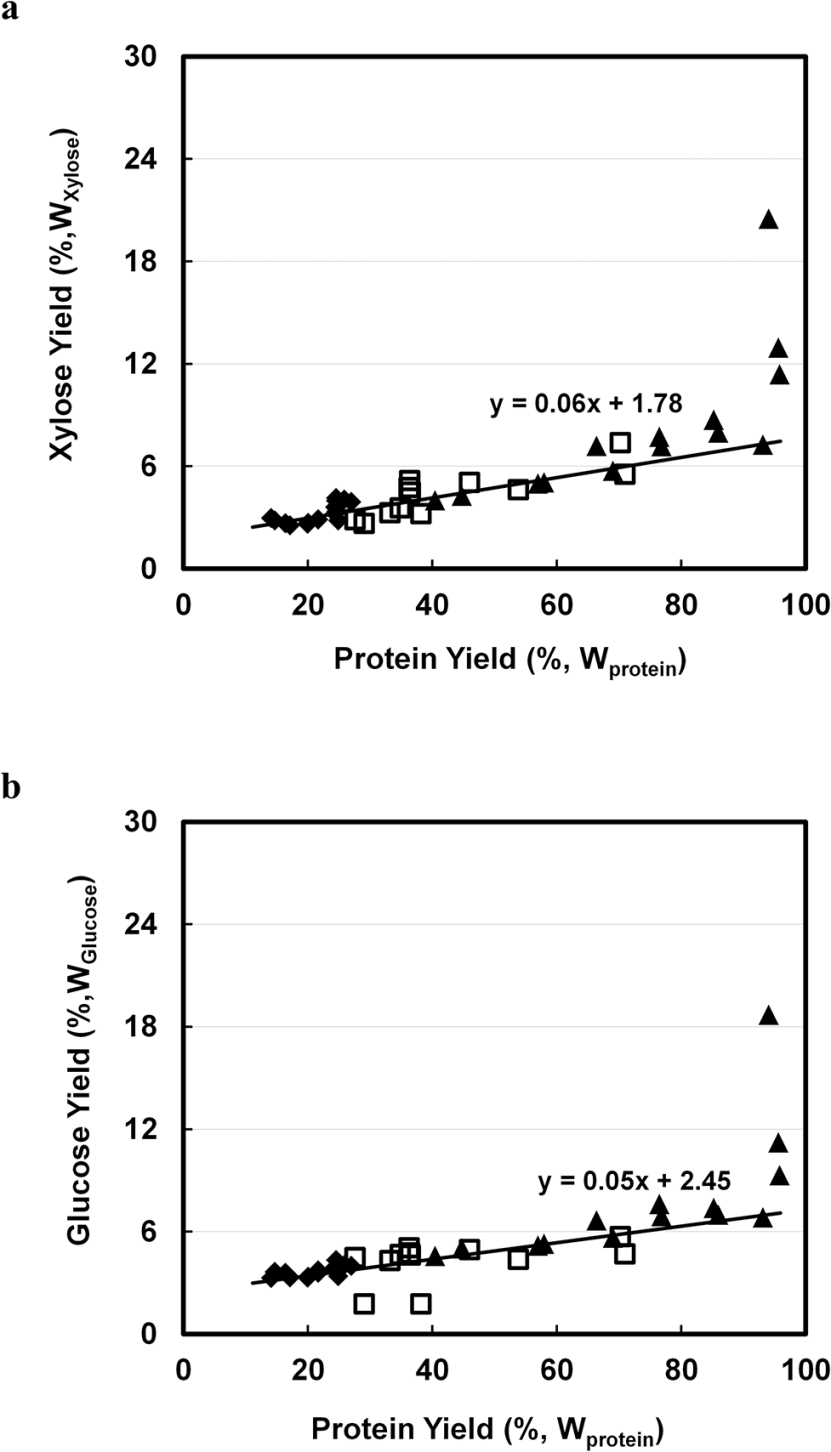
Correlation of extracted cellulose and hemi-cellulose related sugars with extracted protein (%,W_Protein_) by using 0.1M NaOH with 40 v/w at ◆: 25°C; □: 60°C; ▲ 95°C. a. Xylose (%,W_**Xylose**_). b. Glucose (%,W_**Glucose**_).

To analyse the correlation of the solubilised hemi-cellulose and cellulose with protein extraction, yields of xylose and glucose were measured and plotted against protein yields. Generally, yields of xylose and glucose are less than 8% (Fig 4A and 4B) with a highest yield of around 20% only at the harshest condition applied, when all protein had already been extracted. The yield coefficients of these two sugars to protein are therefore relatively low, 0.06 for xylose and 0.05 for glucose. Considering some xylose and glucose may be emanated from side chains of RGI and RGII pectin, the correlation slopes of these two sugars with protein are close to zero. Therefore, high protein yield is probably not due to disruption of hemi-cellulose or cellulose.

### Correlation of non-determined component yields with protein yields

**Fig 5:**
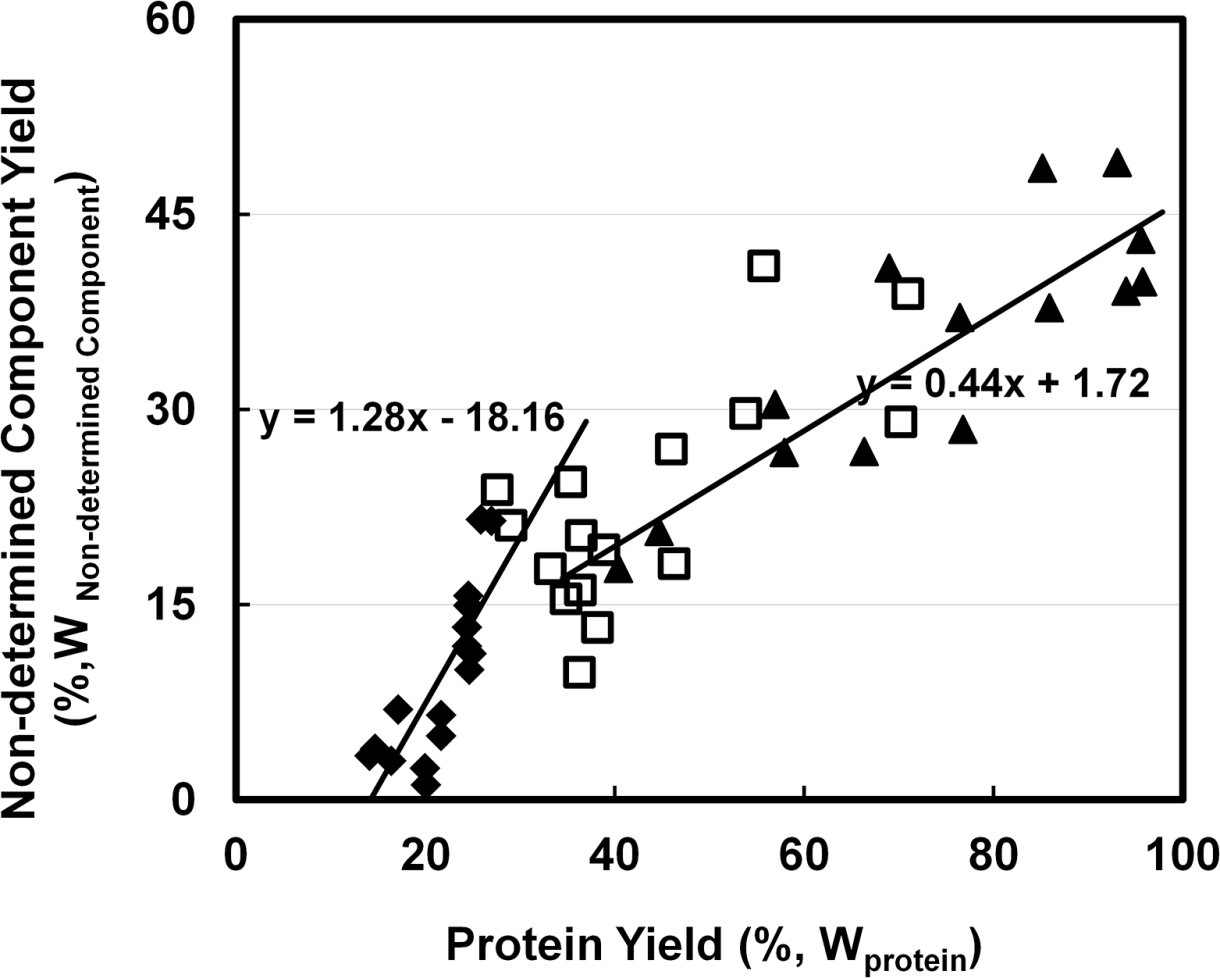
Correlation of extract undetermined components (%,W_Non-determined Components_) with extracted protein (%,W_Protein_) by using 0.1M NaOH with 40 v/w at ◆: 25°C; □: 60°C; ▲ 95°C.

To investigate the influence of lignin on protein extraction, yields of non-determined components (likely to be majorly lignin and lipid) were plotted against yields of protein (Fig 5). Generally, yields of non-determined components were positively correlated to protein yields, but a bend was observed in the curve of Fig 5 and two correlation slopes were calculated. When protein yield was less than 40%, the yield coefficient of non-determined components to protein was 1.28, which was almost 3 times higher as when protein yield was above 40%. The two coefficients might indicate that the yields of two major non-determined components, lipid and lignin, have different correlation with yields of protein, and one of them was completely extracted at mild condition. As lipid can be released with mild alkaline while lignin was insoluble in the same condition [23, 40], lipid possibly contributed to the higher slope while lignin contributed to the lower (0.44). The extractability of lignin showed a maximum of only 25% of total lignin at 95% protein extraction. This means that it is either not necessary to completely dissolve lignin to facilitate protein extraction, or that another component is limiting protein extraction.

## Discussion

### How does alkali aid protein extraction in leaves

Although the role of lignin in protein extraction is still unclear, the correlation of RGI extraction yield with protein extraction yield is clearly shown. In leaf cell wall, RGI is rooted in a putative structure functioning as a scaffold in which galactan and arabinan occur as side chains, forming a kind of molecular brush [41]. It is often correlated with stages of cell development [20] and can covalently-link to xylogulcan (hemi-cellulose) [42, 43], phenolic acids (lignin) [43, 44] and cellulose [45]. The mechanism of how RGI links to other components is still controversial, but how alkaline solutions can extract RGI has been reported [45, 46]. It has been stated that extraction of pectin with side chains of arabinan and galactan is more sensitive to temperature than alkaline concentration [46]. Previous conditions for extracting 80% of arabinose and galactose [46] were similar to our protein extraction condition for 95% protein (0.1M NaOH at 95°C) again suggesting RGI pectin is the key to obtaining high protein yields.

### Integrated biorefinery concept for leaves

An optimized alkaline protein extraction process that is based on our previous work [7]has the lowest chemicals and energy cost with highest protein yield among peer studies [6, 13, 26]. To further reduce the use of chemicals and improve quality of protein, an integrated biorefinery concept is recommendable.

Phenolic components and organic acids in leaves can be removed by solvent extraction [30, 47] in the first step. For example, using ethanol pre-treatment at ambient temperature can extract all caffeine and soluble polyphenols from GTR (data not shown). In the extracts, a yield of 10% N-containing components was detected [7]. These N however was not derived from protein, but from caffeine and chlorophyll (data not shown). This step does not only yield product, but the removal of phenolic compounds also prevents their reaction with protein under alkaline conditions, and may thereby also improve the digestibility of the final protein product [48].

Using relatively mild alkaline conditions (20°C <T<60°C), leaf components, such as HG pectin, RG II pectin, protein and lipids (or organic acids), can be obtained. When all HG pectin (galacturonic acid) was extracted, approximately 32% of N-containing components were also extracted (Fig 3A). Assuming that from this 32%, 10% are non-protein N-containing components extracted by solvent extraction, in this step approximately 22% of protein (25% of total real protein) will be obtained. To reduce alkali consumption, relatively low pH (9–11) is recommended; for excess alkali will only be consumed by the buffering components, such as polyphenol or pectin. The products obtained in this second step can be applied in food industry as functional ingredients, as they are obtained at relatively mild conditions.

When more severe conditions (95°C, pH 13) are applied, the network of RGI pectin and lignin that makes up the cell wall starts to collapse, and membrane protein and proteins from inside the cells begin to liberate. When all protein is extracted approximately 80% of galactose and arabinose (Fig 3), 8% xylose and glucose (Fig 4), and 42% non-determined components (Fig 5) can be extracted. After solvent extraction and subsequent mild alkaline extraction, 68% N-contain components (75% of total real protein), 60% RGI pectin (galactose and arabinose), 5% (hemi-) cellulose, and 25% lignin are obtained. Protein purity will be approximately 55%. Due to this high concentration, these protein products have a high value for application in animal feed.

The remainder, which majorly contains lignocellulose can be hydrolysed at extreme conditions with temperatures above 100°C and alkali concentrations above 0.5M to release mono-sugars [22, 49]. Alternatively, the lignocellulose can be used as a feedstock for producing bio-ethanol, for which alkaline pre-treatment was proved to increase the conversion rate [50].

**Fig 6:**
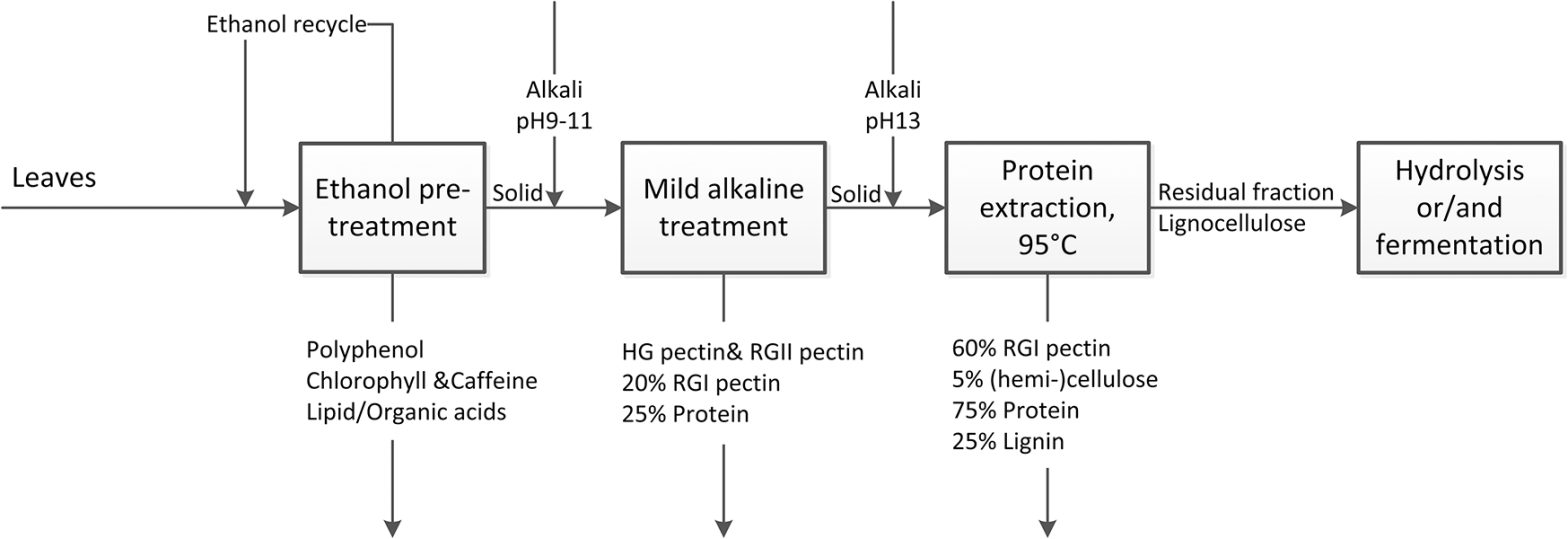
Integrated leaf biorefinery concept. Numbers are estimated based on results with green tea residue (GTR).

Based on the results presented here, and on previous results from literature that aimed for extracting pectin, lignin, hemi-cellulose, and cellulose [22, 46, 49], an integrated leaf biorefinery scheme, which may consist of four steps, can be designed. This is illustrated in Fig 6 with our results on green tea residues.

### Outlook

Next to the presented chemical approach to leaf biorefinery, new approaches can be developed. Using enzymes to specifically break down RGI pectin or mechanical disruption of the epidermal layer in plant cell tissue may improve protein yields in mild conditions such that native protein with higher quantity and quality may be obtained.

## Conclusion

The use of the integrated biorefinery concept under alkaline conditions will add revenue because of the increased value of final products and reduction of production cost. Polyphenol, lipid, pectin, protein and lignocellulose can be obtained separately with higher purity and quality that improves their commercial value as final products or mediates further conversion. The integrated process can reduce alkali consumption for protein extraction, as buffering components such as polyphenol and pectin will be extracted priory. Alkaline treatment can be universally applied to other leaf species [7], such as grass, which brings promising prospects for leaf biorefinery.
